# The tradeoff of solitude? Restoration and relatedness across shades of solitude

**DOI:** 10.1371/journal.pone.0311738

**Published:** 2024-12-05

**Authors:** Morgan Quinn Ross, Scott W. Campbell

**Affiliations:** 1 School of Communication, Oregon State University, Corvallis, Oregon, United States of America; 2 School of Communication, Ohio State University, Columbus, Ohio, United States of America; ECHS: Universidade de Tras-os-Montes e Alto Douro Escola de Ciencias Humanas e Sociais, PORTUGAL

## Abstract

Social interaction and solitude entail tradeoffs. Communicate Bond Belong (CBB) theory holds that social interaction can foster relatedness with others at the cost of social energy, whereas solitude can restore social energy at the cost of relatedness. The current study empirically tests this tradeoff of solitude and its implications for well-being by investigating different degrees of solitude. Less complete degrees of solitude (e.g., no interaction with others) were associated with more relatedness *and* restoration than more complete degrees of solitude (e.g., no interaction, no potential for it, and no engagement with media), speaking against a tradeoff. Solitude was less detrimental for well-being among individuals who perceived it to be associated with higher restoration and relatedness. Yet, this finding was independent of social energy expenditure, challenging CBB theory. Future work should consider motivations for solitude and longitudinal approaches.

## Introduction

The benefits of both social interaction and solitude have been discussed for centuries. The challenge is that they need to be balanced, because experiencing the benefits of one often precludes reaping the benefits of the other. In other words, social interaction and solitude entail tradeoffs. Communicate Bond Belong (CBB) theory recognizes these tradeoffs by proposing that social interaction fosters relatedness with others while expending finite reserves of social energy to do so [1], whereas solitude restores the coffers of social energy [2] while reducing relatedness with others in doing so [3].

**Fig 1:**
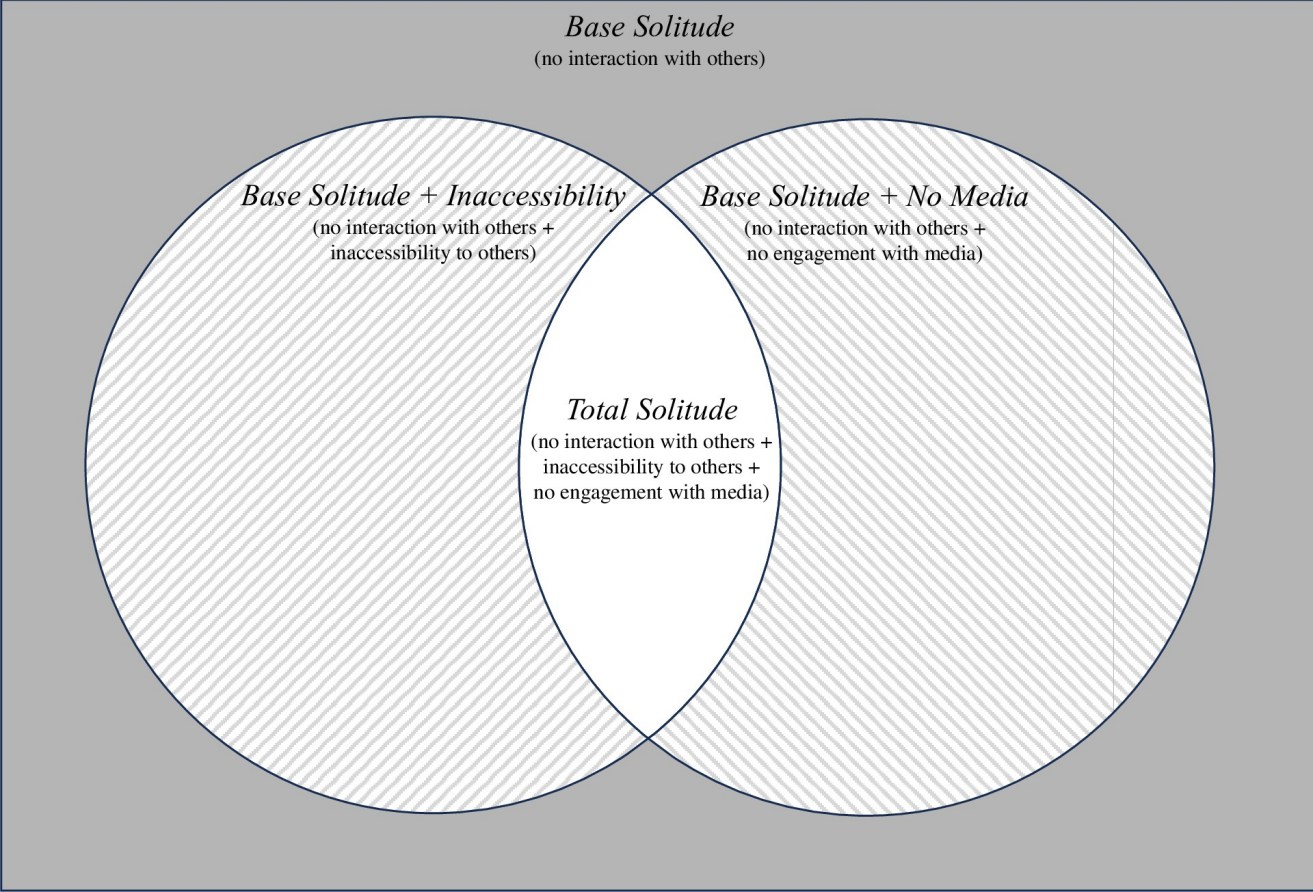
Matrix of solitude. For all shades of solitude, people do not interact with others (across modalities). In Base Solitude, people can remain accessible to others and engage with media. In Base Solitude + Inaccessibility, people are inaccessible to others but can engage with media. In Base Solitude + No Media, people cannot engage with media but can remain accessible to others. In Total Solitude, people are inaccessible to others and cannot engage with media.

The current study tests this tradeoff of solitude by examining conditions under which solitude can be “shaded” by people and technology. [4] argue that accessibility to others and engagement with media can shade the experience of solitude by making time alone more social in nature. We leverage these factors to construct a matrix of solitude (Fig 1), including a *base level* (no interaction with people) and a *total level* (no interaction with people, as well as being inaccessible to others and not engaging with media). This matrix facilitates an investigation of the tradeoff of solitude: experiencing it more completely in total solitude is expected to maximize restoration, whereas experiencing it less completely in base solitude is expected to maximize relatedness.

Further, the current study aims to identify shade(s) of solitude with an optimal balance of restoration and relatedness. According to CBB theory, the key benefit of a balanced tradeoff of social interaction is well-being [1], as people expend energy in the process of gaining relatedness (and ultimately well-being). We expect that this process will be complemented by solitude, when individuals can restore energy while maintaining relatedness.

We begin with an overview of solitude and its shades. We then highlight the tradeoffs of social interaction and solitude, reviewing CBB theory and related work on restoration and relatedness during solitude. After outlining our survey approach, we report the results of confirmatory and exploratory person-level analyses and discuss implications for future work.

### The shades of solitude

Solitude has been traditionally defined as physical aloneness [5]. Against this monolithic view, recent scholarship has proposed that there are shades of solitude, or “key aspects of social context that condition whether and how people experience solitude in daily life” [4]. This proposal was motivated by changes in the (digital) media environment toward greater connectivity. However, factors that condition experiences of solitude have also been identified [6, 7] observed [8, 9], manipulated [10, 11], or self-reported [12, 13] in past work. Studying different shades of solitude addresses heterogeneity in how people experience it, while deepening our knowledge of the contours that give it shape [14].

The current study focuses on two key factors that hinder total solitude: (1) accessibility to others and (2) engagement with media [4]. We select these factors because of their theoretical importance and practical relevance in the digital era, particularly with the rise of mobile communication technology, which uniquely supports anytime-anywhere accessibility to others and media [15]. We define accessibility to others as the potential for social interaction. For example, co-present others and smartphones typically indicate accessibility to others by facilitating potential for social interaction [4]. This study extends prior work that examined inaccessibility to others during solitude but did not contrast it with accessibility [16]. We define engagement with media as indirect communication [4], or any way that the self is “populated” with the perspectives of others [17] besides social interaction. Engagement with media applies to conventional language of “mass media,” but in today’s media environment there are many other opportunities to experience content and communication, without directly interacting with others. This study extends prior work (e.g., [10]) by including analog media, such as books and artwork, as well as digital media, such as television and social media. Notably, some media (e.g., social media) imply accessibility to others and other media (e.g., books) do not.

The baseline for our matrix of solitude is the lack of social interaction with others, in line with recent scholarship on solitude (see [4] for a review). Although not interacting with others is the minimum necessary condition of solitude, it does not capture other layers of social connection that can hinder one from fully experiencing solitude. In particular, having access to others and media keeps people oriented to the social world [17]. We therefore consider inaccessibility to others and no media engagement as conditions that upgrade base solitude into “noncommunication” [4]. Thus, our matrix runs the gamut from *base* solitude (i.e., no interaction with others) to *total* solitude (i.e., no interaction, no potential for it, and no engagement with media), while accounting for shades in between.

This matrix brings focus to the tradeoffs of solitude. In line with the benefits and costs of solitude overall [5], we expect that experiencing solitude in its most complete form can maximize its benefits as well as its costs. The current perspective on the tradeoff of solitude has its roots in CBB theory [1].

### The tradeoff of social interaction

CBB theory [1] argues that social interaction contributes to the fulfillment of the need to belong [18]. Critically, it holds that social interaction is driven by the tradeoff between relatedness and energy. [1] write that, “the result of any given communication episode varies by feelings of relatedness, which is theorized to be a proximal indicator of the need to belong being satiated. Simultaneously, all social interactions expend social energy” (pp. 38–9). Social interaction yields relatedness, indicating fulfillment of the need to belong, but it also taxes finite levels of social energy, which is required for social interaction. The tradeoff emerges because relatedness is positively associated with energy expenditure [19]; social interaction fosters relatedness but depletes energy.

This tradeoff is represented by the relatedness-to-energy ratio in literature on CBB theory (see also closeness-to-energy ratio, [3]; or connection-to-energy ratio, [20]). [3] referred to social interactions with a high relatedness-to-energy ratio as “high in social calories” (p. 396) because they contribute to the fulfillment of the need to belong at a reasonable cost to social energy. Empirically, social interactions with a high relatedness-to-energy ratio reduce the need for subsequent social interaction [3] and are associated with increases in (some) well-being indicators [21]. There are two avenues to maximize the relatedness-to-energy ratio. First, individuals can engage in striving behaviors (e.g., meaningful conversation), which increase both relatedness and well-being. However, striving behaviors come at a higher cost of social energy [21]. Second, individuals can engage in mundane maintenance behaviors (e.g., small talk) to reduce energy expenditure. However, these interactions have minimal impact on relatedness [3].

Notably, the current study asserts that the relatedness-to-energy ratio should be recast as a *difference*. CBB theory argues that relatedness gains should be maximized and energy expenditure should be minimized [1], treating these two competing drives equally. However, it does not argue that the ratio of relatedness to energy *per se* should be maximized. Because energy is the denominator of the relatedness-to-energy ratio, changes in relatedness and energy affect the relatedness-to-energy ratio more at lower (vs. higher) levels of energy—mathematical complexity that goes beyond the parsimonious scope of CBB theory. Yet, it is important to note that the difference and ratio are highly correlated and our findings are robust to either approach (see S1 File).

In response to this tradeoff, individuals seek a homeostatic balance between relatedness and energy ([1], p. 36). Solitude plays a key role in the homeostatic system by allowing individuals to restore social energy [16]. [19] found that individuals desire solitude after energy-intensive social interactions and that individuals who typically engage in such interactions are more likely to experience solitude. Similarly, [22] demonstrate that longer periods of social interaction are followed by longer periods of solitude; after expending more social energy, people need more time to restore it through solitude. This pattern of results aligns with findings that people experience less arousal in moments of solitude [11, 23], suggesting that individuals expend less energy (and possibly restore it) during solitude.

Connecting this tradeoff to well-being, [16] tested whether the restorative potential of solitude facilitates well-being. In the United States, solitude was more positively associated with life satisfaction for individuals who engage in more in-person social interaction, social interaction via mobile media, and self-disclosure via mobile media. These individuals expend more social energy and would thus derive more benefits from restoring social energy through solitude. Our first two hypotheses represent replications of those exploratory findings. For robustness, we leverage multiple shades of solitude (noted above), multiple well-being indices (life satisfaction, affective well-being, and loneliness; [21]) and key striving behaviors from literature on CBB theory (meaningful conversation, affectionate communication, catching up, and joking around; [3]). Additionally, we focus on chosen solitude given its more consistent associations with well-being ([16], see also [24]) and that unchosen solitude is less likely in the contemporary landscape of mediated social connection [25].

**H1:** For individuals who engage in more social interaction, solitude is more positively associated with life satisfaction and affective well-being and more negatively associated with loneliness.
**H2:** For individuals who engage in more striving behaviors, solitude is more positively associated with life satisfaction and affective well-being and more negatively associated with loneliness.

### The tradeoff of solitude

Conversely, solitude facilitates the restoration of social energy at the expense of relatedness with others. Shades of solitude vary in restoration and relatedness, with implications for well-being.

### Restoration

As reviewed above, solitude allows individuals to restore social energy. However, most of this work measures energy restoration indirectly. Empirical work that more directly measures energy restoration primarily stems from attention restoration theory [26, 27]. Attention restoration theory is primarily concerned with how people can restore their attention (i.e., the ability to avoid distraction), such as by taking a walk in nature. Because attention restoration requires regaining the energy needed to avoid distraction, attention restoration partly indexes energy restoration [26, 28], offering traction for the current study. Literature in this vein argues that restoration varies as a function of the environment, with solitude playing a crucial role. In nature, being alone is more restorative than being with company, whereas in urban environments, people find company to be more restorative than solitude [2, 29]. Yet, natural environments are more restorative than urban environments overall, likely due to their lower expectations for social interaction [2]. Moreover, people desire social interaction more when they feel restored [28], linking restoration to subsequent social interaction.

The current study thus expects variation in restoration across environments. The above work suggests that more complete shades of solitude will be more restorative. Accessibility to others involves vigilance to the outside world, fostering stress that inhibits restoration [30]. Although engaging with media can be an immersive and enjoyable means of recovering from fatigue through distraction [31], it seems to undermine restoration by offering a connection to the social world [17], rather than an opportunity to disconnect from it to clear one’s mind and reflect. We therefore expect that accessibility to others and engagement with media reduce restoration. Accordingly, we offer the following hypothesis:

**H3:** Total solitude offers the highest restoration, followed by base solitude + inaccessibility and base solitude + no media, and finally base solitude.

### Relatedness

However, solitude does not facilitate feelings of relatedness with others. Notably, [3] examined how relatedness varied as a function of eleven communication episodes. Not engaging in social interaction (i.e., base solitude) was associated with significantly less relatedness than seven communication episodes, including both striving behaviors and mundane maintenance behaviors. After accounting for the interaction partner, not engaging in social interaction was associated with significantly less relatedness than striving behaviors and similar relatedness as mundane maintenance behaviors. Taken together, relatedness emerges from aspects of our social environments–certain types of interactions (i.e., striving behaviors) and certain interaction partners (i.e., mundane maintenance behaviors with close friends and family)–that are absent from solitude.

[3] only measured base solitude. Yet, we additionally anticipate variation in relatedness across shades of solitude because they also offer different social environments. Prior work indicates that less complete shades of solitude will offer more relatedness. This work, although typically not focused on solitude, suggests that being available to others and engaging with media offer avenues of feeling socially connected, either through the potential for contact [15, 32] or through media content [10, 17]. We therefore expect that accessibility to others and engagement with media support relatedness. Accordingly, we offer the following hypothesis:

**H4:** Base solitude offers the highest relatedness, followed by base solitude + inaccessibility and base solitude + no media, and finally total solitude.

### The restoration-and-relatedness sum

Taken together, we expect that more complete shades of solitude will offer more restoration and less complete shades of solitude will offer more relatedness. In line with prior work on the conditional benefits of solitude [33], we expect that these dynamics have implications for well-being. On the one hand, people should experience higher well-being with more complete shades of solitude, because these shades restore more energy for social interactions. On the other hand, people should experience higher well-being if they engage in less complete shades of solitude, because these shades retain more relatedness with others.

To resolve this tension, we propose the restoration-and-relatedness sum for solitude. Like the relatedness-and-energy difference for social interaction, the restoration-and-relatedness sum treats the competing drives for relatedness and energy equally. Shades of solitude with a high restoration-and-relatedness sum restore social energy while retaining relatedness with others. To maximize the restoration-and-relatedness sum, individuals can maximize restoration or relatedness; however, increasing one will likely decrease the other. Thus, it is unclear which shade will have the highest restoration-and-relatedness sum. We therefore pose the following research question:

**RQ1**: Which shade(s) of solitude will offer the highest restoration-and-relatedness sum?

We then expect that shades of solitude with higher restoration-and-relatedness sums will yield stronger findings for H1 and H2. Social interaction entails people expending energy to gain relatedness (and ultimately well-being). This homeostatic system’s production of well-being should be supported by solitude: people restoring energy while maintaining relatedness. Therefore, individuals who engage in more social interaction or striving behaviors (i.e., expend more social energy) should experience higher well-being if they engage in shades of solitude that restore energy and maintain relatedness.

Thus, the following hypothesis tests a three-way interaction between solitude, social interaction (H1) or striving behavior (H2), and the restoration-and-relatedness sum (we reworded this preregistered hypothesis for clarity while preserving its meaning):

**H5:** The relationships in H1 and H2 will be stronger for shades of solitude that individuals perceive as having a higher restoration-and-relatedness sum.

## Method

### Participants

We recruited 1,469 participants through Cloud Research in August 2023. Participants were excluded if they failed at least one attention check (*n* = 429) or self-reported that their data should be excluded (*n* = 37). We additionally excluded 115 participants who provided starkly inconsistent responses to the measures of solitude frequency: when their response to a more complete shade of solitude was two or more scale points higher than their response to a less complete shade of solitude (not preregistered). Our final sample (*N* = 888) was 61.9 years old on average (*SD* = 14.9) and was comprised of 359 men, 525 women, one transgender man, two gender non-conforming individuals, and one gender-fluid person.

### Procedure

The study was approved by the institutional review board (IRB) at the last author’s previous institution under approval number HUM00237966. After providing written consent, participants completed measures of solitude, social interaction, well-being, and additional measures for a broader project on the mobile media environment. Participants then shared demographic information.

### Measures

Descriptive statistics can be found in Tables 1 and 2. Full item wordings can be found in the S1 File. All multi-item measures were reliable.

**Table 1:** Descriptive statistics of solitude.

									Restoration-and-Relatedness Sum	
Shade of Solitude	Frequency		Restoration			Relatedness				
	*Mean*	*SD*	*Mean*	*SD*	α	*Mean*	*SD*	α	*Mean*	*SD*
Base Solitude	3.42	1.68	4.18	1.31	.93	4.19	1.46	.89	8.37	2.26
Base Solitude + Inaccessibility	2.91	1.60	4.10	1.47	.96	3.18	1.19	.95	7.27	2.32
Base Solitude + No Media	2.81	1.57	4.02	1.50	.96	3.59	1.65	.96	7.62	2.74
Total Solitude	2.47	1.59	3.85	1.69	.97	3.32	1.81	.97	7.18	3.08

**Table 2:** Descriptive statistics of social interaction and well-being.

Construct	*Mean*	*SD*	α
**Social Interaction**			
Frequency	3.36	1.42	
*Striving Behaviors*			
Meaningful Conversation	3.18	0.89	
Affectionate Communication	3.57	1.01	
Catching Up	3.52	0.89	
Joking Around	3.44	0.98	
**Well-Being**			
Life Satisfaction	4.23	1.53	.92
Affective Well-Being	3.66	1.08	.86
Loneliness	2.04	0.60	.90

The measure for affective well-being used a six-point scale and the measure for loneliness used a four-point scale.

#### Solitude

We developed four short descriptions of the shades of solitude based on [4]. The *base solitude* description asked participants to “imagine situations where you choose to not communicate with others (either in-person or through technology).” This condition served as a base upon which the others were developed. The *base solitude + inaccessibility* description added “and not be available to others (in-person or through technology),” whereas the *base solitude + no media* description added “and not engage with media (e.g., news, books, movies, artwork, websites, apps).” The *total solitude* description included the base solitude description and the inaccessibility and no media additions. The shades of solitude were presented in the following order: base solitude; base solitude + inaccessibility and base solitude + no media (at random); and total solitude. The progression was intended to enhance clarity for participants. For each shade of solitude, participants completed three measures: frequency, restoration, and relatedness. We calculated the restoration-and-relatedness sum by adding the means of restoration and relatedness.

*Frequency*. Participants responded to one item (“How often do you experience this type of situation?”) with response options of *Never* (1) to *Once a day or more* (7). We expanded the response options used in [16] based on pilot data that revealed that many participants experienced solitude infrequently. We used a single item here and elsewhere to quickly capture a distinct attribute (frequency) of a distinct behavior (solitude) [34]. We refer to this measure as “solitude” for simplicity.

*Restoration*. Participants completed the recovery scale from [28]. They were prompted to evaluate whether “this type of situation would allow [them] to” experience restoration. Participants responded to seven items (e.g., “renew energy”) with response options of *Not at all likely* (1) to *Extremely likely* (7).

*Relatedness*. Participants completed the relatedness need satisfaction scale from [35]. They were prompted to evaluate whether “in this type of situation, [they] would feel” relatedness. Participants responded to three items (e.g., “close and connected with other people who are important to me”) with response options of *Not at all likely* (1) to *Extremely likely* (7).

#### Social interaction

*Frequency*. Participants responded to one item (“We are interested in situations when you communicate with others (either in-person or through technology). How often do you experience these situations?”) with response options of *Once a week or less* (1) to *About every five minutes* (7). We refer to this measure as “social interaction” for simplicity.

*Striving behaviors*. Participants completed four items in the everyday talk scale from [36]. Each striving behavior (meaningful conversation, affectionate communication, catching up, and joking around) was captured by one item. Participants responded to each item with response options of *Never* (1) to *Very often* (5).

#### Well-being

*Life satisfaction*. Participants completed the life satisfaction scale from [37]. They responded to five items (e.g., “In most ways my life is close to ideal”) with response options of *Strongly disagree* (1) to *Strongly agree* (7).

*Affective well-being*. Participants completed the four-item affective well-being scale from [38] (see [39]). They responded to four items (e.g., “I feel calm and peaceful”) with response options of *None of the time* (1) to *All of the time* (6).

*Loneliness*. Participants completed the 10-item short-form of the loneliness scale from [40, 41]. They responded to ten items (e.g., “I lack companionship”) with response options of *Never* (1) to *Always* (4).

### Analysis plan

For H1-H2, we preregistered linear multilevel regression models that nested shades of solitude within participants. However, there is no within-person variance in well-being, resulting in models that do not converge. Instead, we specified linear regression models for each shade of solitude. The two-way interaction between solitude and social interaction (H1) or solitude and a striving behavior (H2) was specified as the predictor variable and an indicator of well-being was specified as the criterion variable.

For H3, H4, and RQ1, we preregistered repeated-measures one-way ANOVAs. The shade of solitude was specified as the predictor variable and restoration (H3), relatedness (H4), or the restoration-and-relatedness sum (RQ1) was specified as the criterion variable. Pairwise comparisons used Benjamini-Hochberg corrections to account for multiple comparisons (not preregistered).

For H5, we again preregistered linear multilevel regression models that nested shades of solitude within participants, but we faced the same issue as above for H1 and H2. Instead, we specified linear regression models for each shade of solitude. The three-way interaction between solitude, social interaction, and the restoration-and-relatedness sum (H1) or solitude, a striving behavior, and the restoration-and-relatedness sum (H2) was specified as the predictor variable and an indicator of well-being was specified as the criterion variable. We deviated from our preregistered plan by not decomposing the restoration-and-relatedness sum into grand-mean and grand-mean-centered components, in order to avoid using sample statistics (i.e., the grand-mean component) as moderators.

Last, the S1 File on the Open Science Framework page reports a preregistered secondary analysis that was omitted from the main manuscript. We refer to the results of this analysis in the discussion.

## Results

### Confirmatory analyses

We did not find support for H1. Across shades of solitude and well-being indicators, there were no significant interactions between solitude and social interaction.

**Fig 2:**
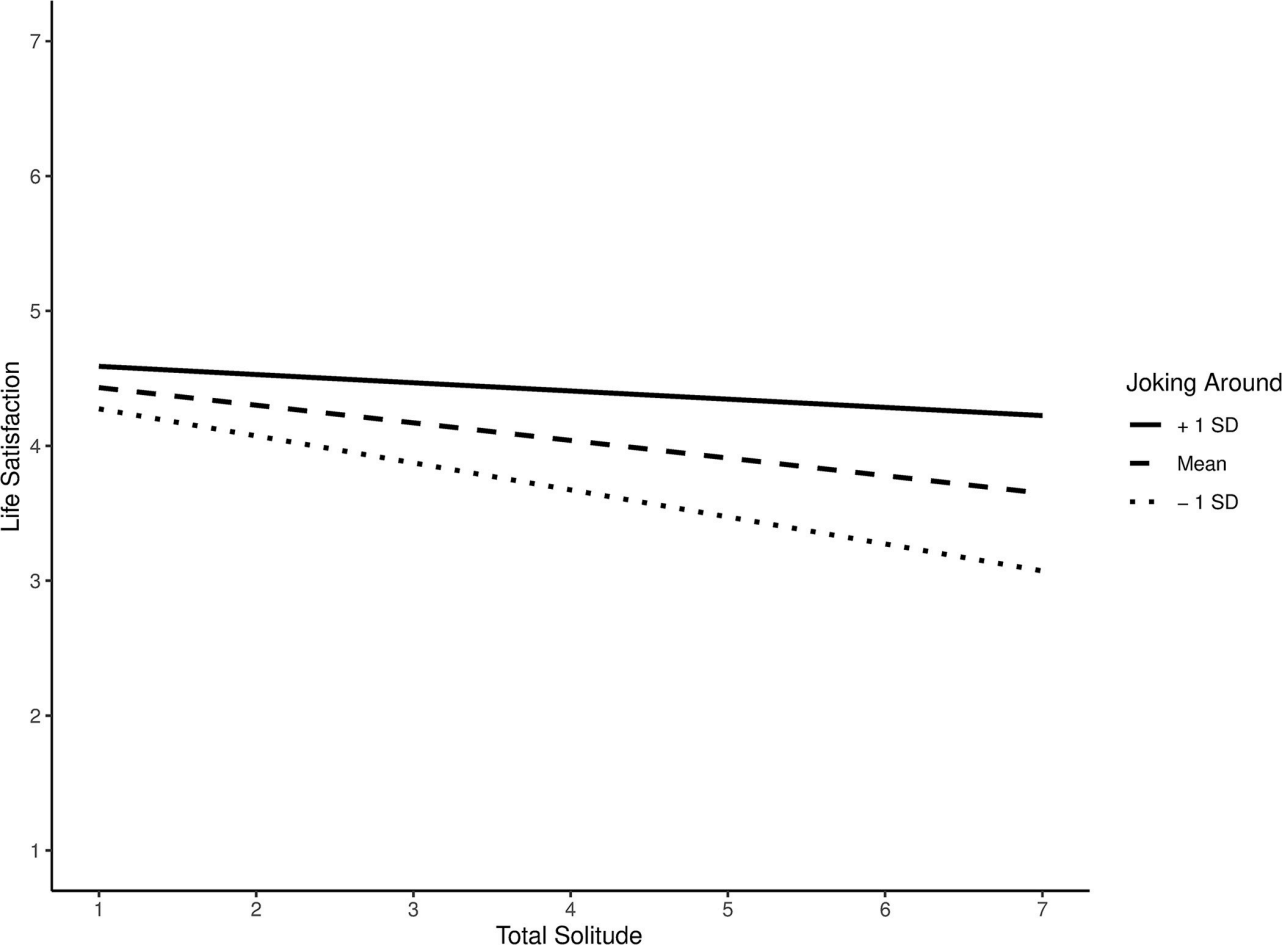
Representative interaction for H2. The striving behavior of joking around weakens the negative relationship between total solitude and life satisfaction.

We found partial support for H2 (summarized in Table 3 and fully reported in the Analysis Scripts on the Open Science Framework page). We report significant interactions for each of the four striving behaviors, all of which were in the expected direction (positive for life satisfaction and affective well-being, negative for loneliness; we report the absolute values of standardized coefficients for clarity). For base solitude + inaccessibility and total shades of solitude across well-being indicators, as well as for base solitude + no media and affective well-being, there was a significant interaction between solitude and meaningful conversation (.23 < *β* ‘s < .41, *p*’s < .04). Across shades of solitude (except base solitude) and well-being indicators (except loneliness for base solitude + no media and total solitude), there was a significant interaction between solitude and affectionate communication (.21 < *β* ‘s < .36, *p*’s < .05). Across shades of solitude and well-being indicators, there were no significant interactions between solitude and catching up. Across shades of solitude (except base solitude) and well-being indicators (except affective well-being), there was a significant interaction between solitude and joking around (.28 < *β* ‘s < .38, *p*’s < .02). Simple slopes indicated that higher levels of striving behaviors weakened the negative relationship between solitude and well-being, with mean + SD levels of striving behaviors occasionally yielding a null relationship between solitude and well-being. Fig 2 presents a representative depiction of this interaction using the *interactions* package in R [42].

**Table 3:** Summary of partial support for H2.

	Striving Behaviors				
	*Meaningful Conversation*	*Affectionate Communication*	*Catching Up*	*Joking Around*	
Solitude					Well-Being
*Base Solitude*	.13	.23	.04	.24	*Life Satisfaction*
	.23	.23	-.03	-.01	*Affective Well-Being*
	-.13	-.12	-.10	-.22	*Loneliness*
*Base Solitude + Inaccessibility*	.32**	.35**	.15	.38**	*Life Satisfaction*
	.36**	.36**	.04	.14	*Affective Well-Being*
	-.27*	-21*	-.10	-.34**	*Loneliness*
*Base Solitude + No Media*	.23	.29*	.21	.29*	*Life Satisfaction*
	.33**	.25*	.06	.05	*Affective Well-Being*
	-.19	-.11	-.17	-.28*	*Loneliness*
*Total Solitude*	.25*	.26*	.15	.29*	*Life Satisfaction*
	.41***	.29*	.07	.11	*Affective Well-Being*
	-.23*	-.15	-.07	-.28*	*Loneliness*

Values indicate standardized coefficients for the interaction terms (solitude * striving behavior) predicting well-being.

*p < .05

**p < .01

***p < .001.

We found evidence against H3. Restoration significantly differed across shades of solitude, *F*(2,190) = 24.0, *p* < .001, but pairwise comparisons indicated that base solitude had the highest restoration (*M* = 4.18), followed by base solitude + inaccessibility (*M* = 4.09), base solitude + no media (*M* = 4.02), and total solitude (*M* = 3.85).

We found partial support for H4. Relatedness significantly differed across shades of solitude, *F*(2,140) = 189, *p* < .001. As expected, pairwise comparisons indicate that base solitude had the highest relatedness (*M* = 4.20), followed by base solitude + no media (*M* = 3.60). However, total solitude was associated with higher relatedness (*M* = 3.34) than base solitude + inaccessibility (*M* = 3.19).

Turning to RQ1, the restoration-and-relatedness sum significantly differed across shades of solitude, *F*(2,100) = 115, *p* < .001. Pairwise comparisons indicate that base solitude had the highest sum (*M* = 8.37), followed by base solitude + no media (*M* = 7.62), and finally base solitude + inaccessibility (*M* = 7.27) and total solitude (*M* = 7.18).

Finally, we did not find support for H5. Across shades of solitude and well-being indicators, there were no significant interactions between solitude, social interaction, and the restoration-and-relatedness sum or between solitude, striving behaviors, and the restoration-and-relatedness sum.

### Exploratory analyses

Although H5 analyses yielded insignificant three-way interactions, they revealed significant two-way interactions between solitude and the restoration-and-relatedness sum. We therefore reran these models without social interaction or striving behaviors (not preregistered). For each combination of the four shades of solitude and the three well-being indicators (12 models), there was a positive interaction between solitude and the restoration-and-relatedness sum (.33 < *β* ‘s < .63, *p*’s < .02). Simple slopes indicated that higher levels of the restoration-and-relatedness sum weakened the negative relationship between solitude and well-being, with mean + SD levels of the restoration-and-relatedness sum yielding a null relationship between solitude and well-being in five of the 12 models.

## Discussion

The current study drew from CBB theory to test whether solitude entails a tradeoff between restoration and relatedness. More complete degrees of solitude were indeed associated with less relatedness (H4), but they were also associated with less restoration (H3). For solitude, it appears that restoration and relatedness do not trade off; rather, they go hand in hand (RQ1). Exploratory analyses indicate that higher restoration and relatedness are associated with less negative relationships between solitude and well-being, illustrating the value of these CBB theory concepts for studying solitude. However, although individuals who expend more social energy were expected to benefit more from solitude that restores energy and maintains relatedness, this was not supported (H5), challenging CBB theory. The lack of support for H1 –and particularly the partial support for H2 –suggest that this challenge could be met by integrating motivations for solitude into CBB theory. Based on these findings, we additionally complicate the roles of restoration and relatedness for CBB theory more broadly.

First, the pattern of relatedness across shades of solitude was mostly in line with expectations: base solitude had the highest relatedness, followed by base solitude + no media, total solitude, and base solitude + inaccessibility (H4). The only deviation from expectations was total solitude having higher relatedness than base solitude + inaccessibility. Since total solitude also involves inaccessibility, this may be due to measurement error: inaccessibility may have been more salient for base solitude + inaccessibility and thus reduced relatedness. However, the additional lack of media engagement during total solitude may indeed facilitate relatedness. Media engagement solitude would represent so-called “passive” usage that does not involve communication with others, which can inhibit relatedness ([11]; but see [43]). Moreover, it is possible that people engage in relatedness-promoting practices (e.g., meditation) in the absence of media [44]. Such practices may be more likely among individuals who engage in solitude, which could explain why this pattern of results held for these individuals but not those who never experienced solitude (see S1 File on the Open Science Framework page).

However, the pattern of restoration across shades of solitude went against expectations: base solitude had the highest restoration, followed by base solitude + inaccessibility, base solitude + no media, and total solitude (H3). These findings suggest that more complete shades of solitude may interfere with restoration. One possibility, albeit speculative, is that more complete shades of solitude are more conducive to imagined interactions, which hold the potential to expend social energy during solitude (see [45, 46]). Additionally, exploratory analyses reveal that restoration did *not* differ across shades of solitude for those who experienced chosen solitude (see S1 File on the Open Science Framework page). In line with these findings, [10] found that boredom is higher for more complete shades of solitude (potentially disrupting restoration), but less so for individuals with higher affinity for being alone (who are more likely to have experienced solitude).

Overall, base solitude had the highest sum of restoration and relatedness, followed by base solitude + no media and then base solitude + inaccessibility and total solitude (RQ1). Base solitude had the highest restoration *and* relatedness, in line with prior work that finds that less complete degrees of solitude are less aversive [10]. Furthermore, perceptions of restoration and relatedness were moderately correlated within shades of solitude (.34 < *r*’s < .56; see Analysis Script on the Open Science Framework page). In addition to boredom [10], low relatedness may be an obstacle to restoration, in line with previous findings that people who have low social support ([47]; see also [21]) may not experience restoration during solitude. Therefore, the current study does not support a tradeoff between restoration and relatedness for solitude.

Yet, exploratory analyses indicate that these CBB theory concepts retain value for studying solitude. Across all combinations of the four shades of solitude and the three well-being indicators (life satisfaction, loneliness, and affective well-being), the sum of restoration and relatedness weakens the negative relationship between solitude and well-being. We suspect that higher levels of restoration and relatedness reflect a positive attitude toward solitude (see [48]). People with a more positive attitude toward solitude may be motivated to experience it due to intrinsic interest, rendering it more beneficial [14]. Indeed, for all shades of solitude, the restoration-and-relatedness sum was higher among participants who experienced solitude (vs. participants who never experienced solitude) (see Analysis Script on the Open Science Framework page). People with a more positive attitude toward solitude are more likely to experience its benefits [49].

However, against expectations, individuals who expend more social energy (via social interaction or striving behaviors) did not benefit more from solitude that restores energy and maintains relatedness (H5). The sum of restoration and relatedness did not moderate H1 or H2. Although the previous results indicate that restoration and relatedness can clarify the relationship between solitude and well-being, they do not clarify how solitude and social interaction are associated with well-being *in tandem*. These null findings challenge the homeostatic balance that is central to CBB theory [16, 19]. Social interaction entails people expending energy to gain relatedness (and ultimately well-being). Solitude should support this homeostatic system’s production of well-being by restoring energy while maintaining relatedness, but that is not the case.

Our findings for H1 and H2 suggest that this challenge to CBB theory could be met by integrating motivations for solitude into CBB theory. We did not find support that social interaction moderates the relationship between solitude and well-being (H1). This null finding persisted across all combinations of the four shades of solitude and our three indicators of well-being. Further, solitude was negatively associated with well-being across levels of social interaction (see Analysis Script on the Open Science Framework page). Both of these findings departed from [16], who found a null association between solitude and well-being overall, but a positive association between solitude and well-being for those who met up with others frequently. One possible reason for this discrepancy is time of data collection: the summer of 2020 (i.e., the height of the Covid-19 pandemic in the United States; [16]) vs. the summer of 2023 (the current study). Balancing solitude and social interaction appears to have been more crucial during a period when in-person social interaction was limited and solitude was either limited or over-abundant (depending on living conditions).

In contrast, the current study found partial support for the moderating role of striving behaviors (H2). The negative association between solitude and well-being was reduced for people who engaged in striving behaviors (meaningful conversation, affectionate communication, catching up, and joking around) more frequently, with variation across indicators. Significant interactions emerged for three of the four striving behaviors (except catching up). Catching up was empirically identified as a striving behavior in [3], but not originally theorized as such [1]; thus, it may be a relatively weak striving behavior. Alternatively, for those who experience more solitude, catching up could be merely instrumental if it is used to reconnect with others after experiencing solitude (see [50]). We found similar results across well-being indicators, albeit slightly more consistent results for life satisfaction (see [21]).

Most notably, significant interactions emerged for three of the four shades of solitude (except base solitude). We also found more consistent support for shades that involved inaccessibility to others. These findings may reflect underlying motivations of solitude. People may seek out solitude–and especially more complete shades of it–when they want to avoid (a) the potential of interacting with other people (due to disliking or anxiety; [51]) or (b) the world at large (through media). However, for people who frequently engage in striving behaviors (vs. social interaction writ large), this motivation may be less likely to be present. CBB theory could be clarified by accounting for such motivations for solitude. Choosing solitude due to negative attitudes toward social interaction may be detrimental to relatedness during solitude and well-being overall, offsetting the benefits of restoration [52]. Conversely, choosing solitude due to positive attitudes toward solitude (as discussed above) would preserve the benefits of restoration (and relatedness maintenance), upholding the homeostatic system [53]. Future research should measure motivations for solitude to test this proposition.

In addition to this proposition, we also complicate the roles of restoration and relatedness in CBB theory. We first consider three possibilities for restoration. First, perceptions of restoration may matter less than the objective potential for restoration. For H2, we found consistent results for more complete shades of solitude, even though they were perceived to be less restorative than base solitude (H5b). Second, our conceptualization and operationalization of restoration may need to be specified. Energy restoration may not capture the restoration of *social* energy specifically. However, [1] do not distinguish between the two. Further, we obtained our measure of restoration from literature on attention restoration. Yet, energy restoration is part of attention restoration [26], the measure was unidimensional, and results were generally robust to operationalizing restoration as the single item that most closely indexes energy restoration (“to renew energy;” see Analysis Script on the Open Science Framework page). Third, and most broadly, it may be useful to move away from the notion that “social interaction expends energy and solitude restores it.” On the one hand, people do not expend much energy in certain social interactions. Further, even if social interaction expends social energy in the short-term, certain individuals (e.g., extroverts) may gain energy from it in the long-term (see [54]). On the one hand, people don’t seem to restore energy that much from (especially more complete) shades of solitude. In fact, engaging in solitude–especially in the current media environment–likely requires energy itself. Future work should consider how solitude can expend energy and social interaction can generate energy.

We consider three possibilities for relatedness as well. Relatedness is an indicator of well-being [1], and it follows that relatedness during solitude would indicate well-being. However, experiencing relatedness in the absence of other people may be maladaptive. Instead, it may be optimal to experience *some* relatedness during solitude; enough to be able to engage in restoration, but not too much to reduce motivation for subsequent social interaction. Relatedness may be especially important for individuals who do *not* frequently engage in striving behaviors–against our hypothesis. These individuals may be more likely to experience loneliness during solitude (see [21]). Future work should explore the directionality and potential curvilinearity of relationships between relatedness during solitude and well-being. Finally, as above, it may be useful to move away from the notion that “social interaction enhances relatedness and solitude reduces it.” Not all social interactions enhance relatedness and not all solitude experiences reduce relatedness (e.g., [44]). Indeed, [3] found that solitude was associated with more relatedness than certain social interactions (i.e., negative talk) after accounting for the interaction partner. Future work should consider how solitude can enhance relatedness and social interaction can reduce relatedness.

The current study had several limitations. It was challenging to capture shades of solitude. Even though we presented shades in order of less to more completeness, many participants provided inconsistent responses for how frequently they experienced each shade; participants would indicate that they experienced a more complete shade more often than a less complete shade, even though the more complete shade was a subset of the less complete shade. Furthermore, we crafted brief descriptions to avoid participants fixating on certain examples of solitude (see also [10]). However, by excluding such examples, participants may not have recalled *any* experiences of solitude, reflected in the high proportion of participants who reported that they never experienced one or more shades of solitude. When we pretested versions of the solitude prompts, most participants were able to recall solitude experiences; however, we did not ask participants to recall these experiences in the main study. Additionally, it may have been challenging for participants to accurately recall restoration and relatedness of these experiences.

Next, our data were cross-sectional, limiting our ability to determine the directionality of our findings. Although we inferred the directionality of certain relationships based on CBB theory, we remained open to alternative directionalities throughout the discussion. As people navigate the continuum of social interaction to total solitude in everyday life–both shaping and shaped by their well-being–future work should apply experience sampling approaches to capture shades of solitude as well as social interaction as they are experienced (or not) in daily life (e.g., [55]). Such longitudinal approaches would further address concerns about participant recall discussed above.

Last, our sample was older than the general population. We are unsure why this was the case. One possibility is that younger adults were more likely to be excluded due to failed attention checks; however, since demographics were reported at the end of the survey, we cannot test this possibility. Our older sample is not a limitation; rather, it is an indication that our results may be more generalizable to older (vs. younger) populations. Yet, prior work has found support for the tradeoffs of social interaction and solitude in a variety of samples, including older adults (e.g., [22]). Indeed, [22] suggest that these tradeoffs may even be more pronounced in older adults, who have likely developed strategies to optimally balance social interaction and solitude over the lifespan. Thus, older adults may represent a liberal test for a tradeoff between restoration and relatedness, strengthening the lack of evidence for this tradeoff in the current study.

## Conclusion

Based on CBB theory, the current study tested the tradeoff of solitude, examining restoration and relatedness across shades of solitude. Yet, more complete shades of solitude were associated with less relatedness *and* restoration, speaking against such a tradeoff. We found that solitude was less detrimental for well-being among individuals who perceived it to be associated with higher restoration and relatedness. However, this finding was independent of social interaction and striving behaviors, challenging CBB theory. Integrating motivations for solitude into CBB theory may clarify how social interaction and solitude are jointly related to well-being. Future work should clarify how and why solitude accompanies social interaction in daily life.

## Supporting information

S1 File (PDF)
