# Supplementary material for: The tradeoff of solitude? Restoration and relatedness across shades of solitude
Source: PLoS One. 2024 Dec 5;19(12):e0311738. doi: 10.1371/journal.pone.0311738 (PMC11620551; doi:10.1371/journal.pone.0311738)
Supplement: S1 File — (PDF) [file pone.0311738.s001.pdf]

The Tradeoff of Solitude? Restoration and Relatedness across Shades of Solitude

Supplemental Materials

The Rarity of Solitude

Pilot data indicated that many participants never experienced one or more of the shades of solitude. Unlike participants who experienced all of the shades of solitude, these participants were unable to draw from their experiences. We therefore preregistered an exploratory analysis of this distinction, which we pose as a research question:

RQ2: Do H3, H4, and RQ1 differ based on whether participants experienced solitude?

Results

For RQ2, we retested H3, H4, and RQ1 for participants who reported that they never experienced at least one of the shades of solitude (hereafter “never-solitude participants”) as well as participants who reported that they experienced every shade of solitude (hereafter “solitude participants”). Indeed, never-solitude participants were almost 40% of the sample (n = 346). The above finding for H3 held for never-solitude participants. However, restoration did not differ across shades of solitude for solitude participants, $F(1,365) = 0.284$, $p = .81$. The above finding for H4 held for solitude participants. However, H4 yielded full support for never-solitude participants; base solitude + inaccessibility had higher relatedness (M = 2.80) than total solitude (M = 2.56). Turning to RQ1, the restoration-and-relatedness sum significantly differed across shades of solitude for both solitude and never-solitude participants, but with slight changes from the overall findings. For solitude participants, the restoration-and-relatedness sum was higher for total solitude (M = 8.18) than base solitude + inaccessibility (M = 7.82), whereas for never-solitude participants, it was higher for base solitude + inaccessibility (M = 6.41) than total solitude (M = 5.62). Further, for never-solitude participants, base solitude + inaccessibility did not differ from base solitude + no media (M = 6.43).
